# Evaluation of the time required to perform three retreatment techniques with dental microscope and ultrasonic activation for removing filling material from the oval root canal

**DOI:** 10.4317/jced.55100

**Published:** 2018-08-01

**Authors:** Tarek-Fahed Alakabani, Vicente Faus-Llácer, Vicente Faus-Matoses

**Affiliations:** 1 PHD student. Department of Endodontics, Faculty of Medicine and Dentistry, University of Valencia, Spain; 2 Tenured professor, Director of the master’s Program in Endodontics. Department of Endodontics, Faculty of Medicine and Dentistry, University of Valencia, Spain; 3 Co-director of the master’s Program in Endodontics. Department of Endodontics, Faculty of Medicine and Dentistry, University of Valencia, Spain

## Abstract

**Background:**

When endodontic treatment fails, retreatment consists of the complete removal of the root canal filling material for thorough cleaning and reobturation. Various techniques are available for the filling removal procedure, which present varying degrees of efficacy, and take a varying length of time to perform. The aim of this study was to compare the time required to carry out reciprocating, rotary, and manual techniques with dental microscope and ultrasonic activation for removing filling material from root canals.

**Material and Methods:**

Ninety-nine extracted single-rooted teeth with straight and oval-shaped canals were selected. The samples were instrumented with ProTaper Gold System up to file F2 and obturated with AH Plus sealer and GuttaCore. The teeth were randomly divided into three groups (n=33) according to the instruments used for removal of filling material: Group (1) Reciproc blue R50 instrument; Group (2) ProTaper Retreatment instruments; Group (3) manual (Hedstrom files and Gates-Glidden drills), under dental microscope at X10 magnification with Ultrasonic retreatment tip. The time required to remove the filling materials was measured with a chronometer. Data were analyzed statistically applying the Kruskal-Wallis test followed by the Mann-Whitney U-test (*p*<0.05).

**Results:**

The time required to remove filling material was significantly shorter in Group 1, followed by Group 2, the slowest being Group 3 (*P*<0.05).

**Conclusions:**

The reciprocating technique was the fastest method for removing root canal filling material.

** Key words:**Endodontic retreatment, hedstrom file, ProTaper retreatment, reciproc blue.

## Introduction

The main objective of endodontic treatment is to maintain the tooth in appropriate form and function. But occasionally, endodontic treatment fails. In these cases, retreatment must be performed, which involves complete removal of the root canal content to access the apical foramen for thorough cleaning and reobturation (1,2).

Various retreatment techniques have been proposed for regaining access to the root canal system by removing the original filling, using rotary and reciprocating kits, hand files, and ultrasonic tips (3-7).

Some systems were developed only for retreatment root canals. One of these systems is the ProTaper Universal Retreatment System (Dentsply Maillefer, Ballaigues, Switzerland) that consists of 3 instruments for use in each third of the root canal: D1 - 30/.09; D2 - 25/.08; and D3 - 20/.07, these files have convex triangular cross sections similar to those the ProTaper shaping and finishing files (8).

Recently introduced onto the market, Reciproc Blue (RPC Blue; VDW, Munich, Germany) is new-generation single-file system that acts with a reciprocal motion, the latest version in the Reciproc range (RPC, VDW, Germany). As a Reciproc file, Reciproc Blue has an S-shaped cross section, a non-cutting tip, and two cutting edges. The file is manufactured by altering the molecular structure through a new heat treatment in order to increase cyclic fatigue resistance; this heat treatment gives the file its blue color (9).

Several studies show that the use of an ultrasonic tips and dental operating microscope removes the filling material from root canal system more effectively (10,11).

A new core-carrier system has recently become available on the market. Developed by the manufacturers of GuttaCore, the system is easy to remove using rotary files and has a low elasticity modulus, fracturing easily under torsional loading (12).

In addition to the efficiency of the instruments and procedures used in retreatment, the total operating time is another factor conditioning the clinical efficiency of gutta-percha removal techniques; this is the time taken to reach the working length and, ideally, to achieve complete removal of the obturation material (13).

Several studies showed that the reciprocating technique was faster method for removing gutta-percha and sealer than the rotary technique (6,14). on the other hand, other studies showed that the time of removing filling material of reciprocating and rotary systems is similar (4,15,16), whereas, when comparing with hand instrumentation, the hand file is associated with longer retreatment times than filling removal using rotary or reciprocating systems (4,17,18).

The purpose of this study was to compare the time required to remove filling material using three retreatment systems with dental microscope and ultrasonic activation: Reciproc blue R50, ProTaper retreatment, and Hedstrom file with Gates Glidden drills, used for the removal of filling materials from oval shaped canals.

## Material and Methods

-Specimen Preparation 

After the approval of the Ethics Committee with Nº H1512122849636, ninety-nine extracted single rooted teeth for periodontal reasons were selected. Each tooth fulfilled the following criteria: one single-rooted tooth with oval shaped canal with the same curvature <10º according to Schneider’s classification (19), completely developed apices, absence of root filling material, resorption or internal calcifications. specimens were digitally radiographed in mesiodistal and buccolingual direction. Oval shaped canals were defined, when the buccolingual diameter having a maximum diameter of up to 2 times greater than the mesiodistal diameter at 5 mm from the apex according to Jou *et al.* (20).

calculus and Soft tissue were removed mechanically from the tooth surfaces of 99 selected specimens. the teeth were decoronated using a diamond disk to obtain a root length of 17 mm with a working length (WL) of 16 mm.

-Root Canal Preparation and Filling 

Endodontic instrumentation was performed by using the ProTaper Gold system up to instrument F2 (Dentsply Maillefer) and were operated with an X-Smart plus motor (Dentsply Maillefer) at a speed of 300 rpm, following the manufacturer’s recommendations.

During instrumentation, canals were irrigated with 2 mL sodium hypochlorite (2.5%) (Dentaflux, Madrid, Spain) between each file. After completion of preparation, the final irrigation was performed with 2 mL EDTA (18%) (Ultradent, South Jordan, USA) for 2 minutes and 5 mL NaOCL (2.5%). After that, canals were dried using absorbent paper points (Dentsply Maillefer).

 All canals were obturated using a size 25 obturator. After coating the root canal walls with AH Plus sealer (Dentsply De Trey, Konstanz, Germany), the softened obturator was heated in a Therma Prep plus oven (Dentsply Maillefer) and inserted slowly to the working length. The carrier was twisted off, and the GuttaCore was compacted in the orifice of the canal, following the manufacturer’s instructions.

Teeth were digitally radiographed to ensure quality of the root fillings and absence of voids in the obturation of the root canal. The teeth were stored in 100% humidity at 37°C for 30 days to allow the sealer to fully set.

-Root canal retreatment

After one month, the 99 samples were randomly divided into three groups (n=33), according to the filling removal technique:

Group (1) Reciproc technique (Reciproc blue R50): The obturation material was removed using Reciproc blue file R50 (VDW GmbH, Munich, Germany). The file was inserted into the canal and worked in an in and out pecking motion with an amplitude of about 3 mm in the ‘‘RECIPROC ALL’’ mode, applying light apical pressure as recommended by the manufacturer and using an X-Smart plus motor. This procedure was repeated until the instrument reached the WL.

• Group (2) Rotary technique (ProTaper Universal Retreatment): the obturation material was removed using ProTaper Retreatment files (Dentsply Maillefer). Instrument D1 was removed the filling from the cervical third of the canal, whereas instruments D2 and D3 were used in the middle and apical thirds of the root canals, respectively. The instruments were used with an X-Smart Plus motor with 500 rpm speed, applying a crown-down technique. After reaching the working length, D1, D2, and D3 instruments of ProTaper retreatment with brushing action were applied against the canal walls.

• Group (3) Manual technique (Hedstrom files with Gates-Glidden drills): the obturation material was removed from the coronal and middle thirds with sizes 3 and 2 Gates-Glidden drills (Dentsply Maillefer), followed by Hedstrom files (Dentsply Maillefer) of sizes 35, 30 and 25 to remove the root filling material from the apical third with a circumferential quarter-turn push-pull motion until the full working length was reached with a size 25 H file.

The same irrigation protocol was applied in three group. The final irrigation was performed with 2 mL EDTA (18%) and a final rinse with 5 mL sodium hypochlorite (2.5%).

**Figure 1:**
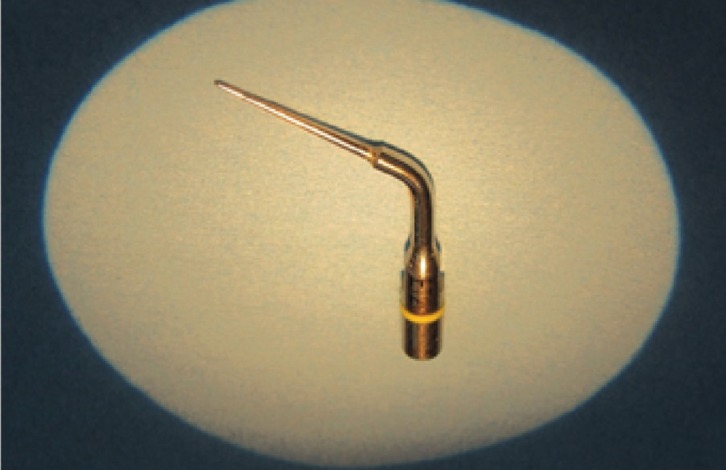
Ultrasonic retreatment tip Et-20.

The ultrasonic retreatment tip Et-20 (size 20, 0.06 taper; no cutting blades) (Satelec Acteon Products, Bordeaux, France) was used for the removal of root canal filling material, applying it three times for 20 second (Fig. 1).

A single operator experienced in three systems performed all endodontic procedures.

Complete removal of the obturation material was defined by the following criteria: when there was no evident filling material on the instrument flute; and when the presence of GuttaCore was no longer visible on the canal walls with a clinical microscope under 10X magnification.

-Time required for gutta-percha removal 

A chronometer was used to measure net time; the total time required to remove the GuttaCore and AH Plus was considered to be the time lapsed from the moment the files were first inserted into the root canal until the files reached the WL. The chronometer was stopped, whenever the instrument was removed from the root canal.

-Statistical analysis

The time required to remove the filling materials was expressed in minutes. The data were examined for normal distribution (Kolmogorov-Smirnov test) and homogeneity of variance (Levene’s test). Non-parametric tests were used to compare the time required to remove the filling materials between groups: Kruskal-Wallis followed by the Mann-Whitney U-test. The level of significance was set at *P* < 0.05.

## Results

Mean ± SD, maximum and minimum values of the time required to remove filling material from Root Canals using the three different retreatment techniques are shown in  Table 1.

**Table 1:**
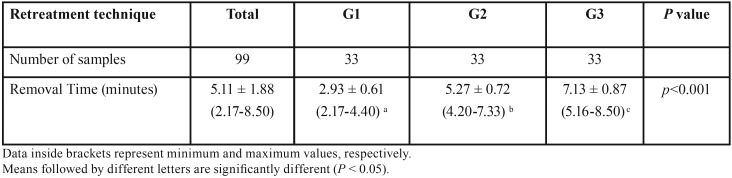
Mean ± SD, maximum and minimum values of the time required to remove filling material (minutes) in Root Canals using three retreatment techniques.

The time required to remove GuttaCore and AH Plus was significantly shorter in the Reciproc blue R50 group (Group 1), than the ProTaper R group (Group 2), and the manual technique using Hedstrom files and Gates-Glidden drills (Group 3) (*p*<0.001).

## Discussion

Removal of root canal filling material is a procedure of major importance in endodontic retreatment because, through the use of instruments and irrigating solutions, it constitutes an effective measure against the debris and microorganisms associated with apical periodontitis (5,21).

In the present study, teeth with oval shaped canals and curvatures <10º were selected, and the initial root canal treatment procedures were the same for all specimens in order to standardize the samples as far as possible. This helped to make reliable intergroup comparisons.

A dental operating microscope and ultrasonic tips were used as these have been shown to improve root canal retreatment (10,11).

GuttaCore was used as the filling material because it has been demonstrated that this material is easy to eliminate with rotary systems (12). Three different root canal retreatment techniques were compared: reciprocating, rotary, and hand files to determine which was the fastest of the three.

According to the results obtained, the reciprocating technique (Group 1) removed filling material in a shorter time than the rotary technique (Group 2) These results concur with previous studies (6,14) and may be attributed to the reciprocating technique’s single-file concept.

In another study, showed that the PTR NiTi rotary system was faster than the reciprocating technique (22). These opposing findings may be explained by variables related to the operator and different methods to calculate the total retreatment time.

The results of the present study revealed that the rotary and reciprocating techniques were faster than the manual technique, findings that concur with previous studies (6,17,18). These findings may be explained by the design of the rotary and reciprocating system (motion, flute design, different taper and active tip). In addition, the softening or plasticization of gutta core caused by the higher rotational speeds, which leads to easier removal of the obturation material (2,23,24).

## Conclusions

Within the limitations of the study, the reciprocating technique was the most rapid method for removing filling material in the retreatment of oval-shaped root canals.
